# Risk Factors for Upper Extremity Impairment after Mastectomy: A Single Institution Retrospective Review

**DOI:** 10.1097/GOX.0000000000005684

**Published:** 2024-07-24

**Authors:** Hannah M. Carr, Ronak A. Patel, Maureen R. Beederman, Nicholas H. Maassen, Summer E. Hanson

**Affiliations:** From the * Section of Plastic and Reconstructive Surgery, Department of Surgery, University of Chicago Medicine and Biological Sciences Division, Chicago, Ill.; † Department of Orthopedic Surgery and Rehabilitation, University of Chicago Medicine and Biological Sciences Division, Chicago, Ill.

## Abstract

**Background:**

Patients with breast cancer treated with mastectomy are more likely to develop upper extremity dysfunction compared with those treated with breast-conserving therapy. This study aimed to identify cancer and treatment characteristics that may be risk factors for development of upper extremity dysfunction in patients treated with mastectomy.

**Methods:**

The authors performed a retrospective chart review of patients at the University of Chicago who were treated with a unilateral or bilateral mastectomy from 2010 to 2020 and developed upper extremity dysfunction based on International Classification of Disease-10 codes. Patients were analyzed by side of body (left or right). Patient demographics and treatment characteristics were extracted from the electronic medical record.

**Results:**

In total, 259 patients met criteria and were included in our study. A total of 396 upper extremities were recorded as experiencing dysfunction and were analyzed. Mean age was 60 years (range = 28–96), and mean body mass index was 28.4 (SD = 7.5). An estimated 54% of patients underwent breast reconstruction. After multivariable analysis, chronic upper extremity pain was found to be associated with ipsilateral radiotherapy (*P <* 0.001) and ipsilateral in situ cancer (0.041). Limited range of motion was found to be associated with ipsilateral invasive cancer (*P =* 0.01), any ipsilateral mastectomy surgery (*P <* 0.001), and ipsilateral radiotherapy (*P =* 0.03). Musculoskeletal dysfunction was found to be associated with no ipsilateral modified radical mastectomy (*P =* 0.033). No oncological or treatment characteristics were found to be associated with decreased strength or adhesive capsulitis. Furthermore, breast reconstruction (implant or autologous tissue based) was not associated with upper extremity dysfunction.

**Conclusion:**

Breast cancer characteristics and treatment modalities may predispose patients treated with mastectomy to developing types of upper extremity dysfunction.

**Takeaways**

**Question:** Are there risk factors associated with upper extremity impairment after mastectomy for breast cancer treatment or prevention?

**Findings:** Treatment factors including sidedness of surgery and radiation therapy were predictors of upper extremity impairment; however, type of reconstruction was not associated with higher rates of pain or dysfunction.

**Meaning:** Breast reconstruction does not put patients at a higher risk for upper extremity impairment after mastectomy, but axillary clearance and radiation do; therefore, consideration should be given to early mobility during and after recovery.

## INTRODUCTION

Breast cancer is the most common cancer among women in the United States, with approximately 13% of women diagnosed with breast cancer during their lifetime.^1^ With improved screening and treatment, the mortality rate of breast cancer is 2.5%.^1^ Surgical treatment, which includes breast-conserving surgery or mastectomy, is integral to breast cancer care, and an increasing proportion of women are choosing to undergo mastectomy over breast-conserving surgery^2,3^ or are pursuing prophylactic mastectomy surgery.^4^ Given the efficacy of surgical, medical, and radiation therapy for breast cancer, there are a large number of women living with the resulting morbidity that interferes with posttreatment quality of life.

Patients who undergo any breast cancer operation report upper extremity symptoms, including pain, stiffness, and functional limitations.^5,6^ Objective evaluations of patients after breast cancer surgery have shown decreased shoulder range of motion (ROM)^6^ and upper extremity strength,^6,7^ atrophy of the pectoralis major and minor muscles,^5^ and diminished muscular response on electromyography.^5^ Additional studies have demonstrated mastectomy to place patients at a higher risk of upper extremity dysfunction than breast-conserving surgery.^8–12^ Patients who have undergone mastectomy are shown to have reduced glenohumeral joint ROM, increased scapular dyskinesis, and force imbalance across the shoulder.^11^ Prolonged upper extremity pain,^9^ limited ROM,^10,12^ and adhesive capsulitis,^13^ a chronic condition characterized by pain and multidirectional limited ROM in the shoulder, are more common in mastectomy-treated patients compared with those undergoing breast conservation.

Still, there is little understanding of factors contributing to upper extremity dysfunction in patients treated with mastectomy. In studies of patients treated with any oncological breast surgery, chemotherapy has been found to be associated with arm/shoulder pain,^14,15^ limited ROM,^12,14^ decreased strength,^16^ and functional disability.^12,14^ Radiation has been shown to be associated with reduced pectoral and rotator cuff muscle volume,^17,18^ bilateral limited ROM,^19^ and overall impairment of the upper extremity.^20^ Axillary surgery^14,15^ and, specifically, number of lymph nodes dissected^9^ are associated with upper extremity pain and limited ROM as well. It is unclear if these relationships between treatment and dysfunction are additive in the mastectomy-treated breast cancer population. A better understanding of factors that may predispose mastectomy-treated patients to further upper extremity sequelae will allow for better patient counseling and rehabilitation pre- and postmastectomy. This study aimed to identify patient, disease, and treatment characteristics associated with upper extremity dysfunction in patients treated with mastectomy.

## METHODS

The authors conducted a single-institution, retrospective chart review of patients at the University of Chicago Medical Center. This study was reviewed and approved by the University of Chicago institutional review board (approval no.: IRB21-0253).

### Patient Selection

Patients were first identified as having undergone mastectomy based on common procedural technique codes between January 1, 2010 and January 1, 2020. Mastectomy nipple and skin-sparing, mastectomy simple bilateral, mastectomy skin sparing, and mastectomy simple procedures were used to identify potential candidates. There were 1202 patients identified from this search. This list was then searched for any members with associated diagnosis codes related to upper extremity dysfunction, including pain in the arm or shoulder, weakness of the upper extremity, neuropathy, disorder of the tendon or synovium, adhesive capsulitis, and unspecified disorder of the limb. Inclusion criteria were age at least 18 years at the time of mastectomy and having received either a bilateral or unilateral mastectomy at our medical center with documentation of any upper extremity dysfunction beyond the 90-day period from the mastectomy operation. Patients were excluded from the study if they did not undergo mastectomy surgery or if upper extremity dysfunction was due to unrelated traumatic injury or was identified before breast cancer treatment. Patients were further excluded if the upper extremity complaint was specifically related to lymphedema of the affected side or chemotherapy-related neuropathy.

The electronic health record was then searched to gather the variables of interest, including invasiveness of cancer (in situ, invasive), type of mastectomy [any, skin-sparing, nipple-sparing, modified radical mastectomy (MRM)], axillary lymph node procedures (any, sentinel node biopsy, axillary node dissection), chemotherapy (neoadjuvant, adjuvant), radiotherapy, reconstruction (any, implant, autologous), prior lumpectomy, completion mastectomy, and indication for completion mastectomy (positive margins, recurrence). Upper extremity dysfunction cohorts include pain, limited ROM as reported by the patient or documented during a physical therapy assessment, decreased strength, adhesive capsulitis, musculoskeletal dysfunction (eg, tendinopathy, rotator cuff pathology), nerve compression distinct from neuropathy, and other upper extremity dysfunction not otherwise specified. The charts of patients meeting our inclusion criteria were reviewed to identify patient-reported symptoms, objective measures of motion and strength from physical therapy or orthopedic evaluation, and clinical diagnoses of upper extremity pathology. Subjects were often included in multiple dysfunction cohorts for analysis if it was documented that they experienced multiple types of dysfunction.

### Data Analysis

Analysis was performed on the patient level and laterality. Only the side of the body experiencing upper extremity dysfunction was included, regardless of surgical laterality. Logistic regression was used to analyze membership of cohorts of dysfunction.^20^ A univariable analysis was conducted to assess variable association with each cohort of upper extremity dysfunction, and subsequent odds ratios (OR) and *P* values were calculated. Variables with a *P* value less than 0.05 were considered significant for the multivariable model. Both univariable and multivariable models were corrected with random intercepts to account for nonindependence of two sides of the same patient.^21^ The strength of the associations was expressed as OR with 95% CIs. A *P* value of less than 0.05 was considered statistically significant.

## RESULTS

### Patient Demographics

In total, 259 patients were identified as having undergone a mastectomy procedure between January 1, 2010 and January 1, 2021 and experienced nontraumatic upper extremity dysfunction, with 396 total extremities recorded as experiencing dysfunction. The median patient age was 60 years with a range of 28–96 years. The average body mass index was 28.4 kg per m^2^ ± 7.15 kg per m^2^. In total, 254 (98%) patients were women, and five (2%) patients were men. Of the total patients, 105 (41%) patients were White, 128 (49%) patients were Black, 14 (5%) patients were Asian or Mideast Indian, and 12 (5%) patients identified as being of multiple races or of a different race. The average follow-up after mastectomy was 33 months. Patient characteristics are summarized in Table 1.

**Table 1.** Patient Characteristics

Characteristic	No. (*N* = 259)	Percent
Age, y		
Median	60	
Range	28–96	
BMI, kg/m^2^		
Mean	28.4	
SD	7.15	
Sex		
Male	5	2
Female	254	98
Race		
White	105	41
Black	128	49
Asian or Mideast Indian	14	5
Multiple races or other	12	5
Ethnicity: Hispanic	8	3
Cancer invasiveness		
In situ	115	44
Invasive	200	77
Mastectomy type		
Skin sparing	213	82
Nipple sparing	34	13
MRM	25	10
Unilateral	154	59
Bilateral	105	41
Prior lumpectomy	59	23
Completion mastectomy	47	18
Positive margins	20	8
Recurrence	22	8
Axillary node procedure	232	90
SLNB	184	71
ALND	80	31
Chemotherapy	153	59
Neoadjuvant	109	42
Adjuvant	75	29
Radiotherapy	127	49
Reconstruction	141	54
Autologous	94	36
Implant	49	19
UE dysfunction		
Pain	220	85
Limited ROM	175	68
Decreased strength	83	32
Adhesive capsulitis	21	8
MSK dysfunction	73	28
Nerve compression	20	8
Other	44	17
Follow-up time, mo		
Median	33	
Range	0–137	

ALND, axillary lymph node dissection; BMI, body mass index; MSK, musculoskeletal; SLNB, sentinel lymph node biopsy; UE, upper extremity.

### Oncological Characteristics

The majority of patients (200, 77%) were diagnosed with invasive breast cancer, whereas 44% (115) had an in situ component. A total of 154 (59%) patients underwent a unilateral mastectomy, and 105 (41%) patients underwent bilateral mastectomies. Skin-sparing was the most common type of mastectomy (232, 90%), whereas 34 (13%) patients underwent nipple-sparing mastectomy, and 25 (10%) patients underwent an MRM. Fifty-nine patients (23%) had a prior lumpectomy before undergoing mastectomy. Of those who had a completion mastectomy, half were due to positive margins on lumpectomy specimen, and half were due to recurrence after completion of treatment. In total, 184 patients (71%) underwent sentinel node biopsy and 80 patients (31%) underwent axillary node dissection. An estimated 109 patients (42%) received neoadjuvant chemotherapy, 75 (29%) patients received adjuvant chemotherapy, and 127 patients (49%) received radiation therapy. Breast reconstruction occurred in 141 (54%), with the majority receiving autologous tissue flaps (n = 94, 36% of total population) compared with implant reconstruction (n = 49, 19% of total).

### Upper Extremity Dysfunction

Of the 259 patients who met inclusion criteria of both any type of mastectomy and any documented upper extremity dysfunction, there were 364 mastectomies and 396 affected extremities experiencing one or more symptoms. On the patient level, 85% (n = 220) of patients experienced upper extremity pain, 68% (n = 175) of patients experienced limited ROM, 32% (n = 83) of patients experienced notable decreased strength, 8% (n = 21) of patients were formally diagnosed with adhesive capsulitis, 28% (n = 73) experienced musculoskeletal dysfunction, and 8% (n = 20) reported compressive neuropathy symptoms distinct from chemotherapy-related neuropathy.

### Upper Extremity Pain

Univariable analysis found upper extremity pain to be associated with the following variables: any ipsilateral cancer, ipsilateral invasive cancer, ipsilateral in situ cancer, any ipsilateral mastectomy, any ipsilateral axillary lymph node procedure, and ipsilateral radiation (all *P <* 0.05). Multivariable analysis of these variables identified ipsilateral radiation [OR 3.81, confidence interval (CI) 2.03–7.16, *P*<0.001) to be associated with long-term upper extremity pain. These results are shown in Table 2.

**Table 2.** Univariable and Multivariable Analysis of Pain following Mastectomy

Characteristic	Univariable			Multivariable		
	OR	OR 95% CI	*P*	OR	OR 95% CI	*P*
Any cancer	2.27	(1.43–3.62)	<0.001			
In situ	1.75	(1.00–3.07)	0.05	1.11	(1.04–2.39)	0.8
Invasive	1.78	(1.11–2.86)	0.017			
Any mastectomy	2.43	(1.42–4.17)	<0.001			
Any node procedure	2.27	(1.43–3.60)	<0.001			
Radiation	3.81	(2.03–7.16)	<0.001	3.81	(2.03–7.16)	<0.001
Preoperative radiation	8.91	(1.19–66.5)	0.033			
Postoperative radiation	2.65	(1.41–4.99)	0.003			
Any reconstruction	1.42	(0.83–2.45)	0.2			

### Upper Extremity Limited ROM

Univariable analysis found upper extremity limited ROM to be associated with any ipsilateral procedure (cancer, mastectomy, or axillary lymph node procedure). In addition, implant-based reconstruction, neoadjuvant chemotherapy, and radiation were associated with limited ROM. Bilateral mastectomies were associated with limited ROM but not more significantly impaired unilateral. Multivariable analysis identified ipsilateral invasive cancer (OR 2.69, CI 1.26–2.57, *P =* 0.01), any ipsilateral mastectomy (OR 5.46, CI 2.12–14.1, *P <* 0.001), and ipsilateral radiation (OR 2.43, CI 1.09–5.43, *P =* 0.03) to be associated with upper extremity limited ROM. These results are shown in Table 3.

**Table 3.** Univariable and Multivariable Analysis of Limited ROM following Mastectomy

Characteristic	Univariable			Multivariable		
	OR	OR 95% CI	*P*	OR	OR 95% CI	*P*
Any cancer	7.04	(3.35–14.8)	<0.001			
Invasive cancer	6.87	(3.35–14.1)	<0.001	2.69	(1.26–5.72)	0.010
Any mastectomy	12.4	(4.95–30.9)	<0.001	5.46	(2.12–14.1)	<0.001
MRM	5.27	(1.79–15.5)	0.003			
Bilateral surgery	2.32	(1.39–3.85)	0.001			
Any node procedure	5.80	(2.96–11.4)	<0.001			
ALND	4.11	(1.81–9.32)	<0.001			
Implant reconstruction	2.67	(1.22–5.81)	0.014			
Neoadjuvant chemotherapy	2.16	(1.27–3.68)	0.005			
Radiation	6.02	(2.72–13.3)	<0.001	2.43	(1.09–5.43)	0.030

ALND, axillary lymph node dissection; MRM, modified radical mastectomy; ROM, range of motion.

### Decreased Strength

Subjective, persistent, decreased strength was associated with ipsilateral cancer, mastectomy, axillary node dissection (but not sentinel lymph node biopsy), or radiation treatment, with univariable analysis only, as shown in Table 4.

**Table 4.** Univariable and Multivariable Analysis of Decreased Strength following Mastectomy

Characteristic	Univariable			Multivariable		
	OR	OR 95% CI	*P*	OR	OR 95% CI	*P*
Ipsilateral cancer	1.8	(1.18–2.74)	0.006			
In situ cancer	1.2	(0.74–1.87)	0.5			
Invasive cancer	1.62	(1.09–2.41)	0.016			
Any mastectomy	2.55	(1.37–4.73)	0.003	2.47	(1.33–4.61)	0.004
Any node procedure	1.82	(1.20–2.76)	0.005			
ALND	1.81	(1.03–3.18)	0.04			
Radiation	1.92	(1.23–2.99)	0.004	1.30	(0.76–2.21)	0.3
Postoperative radiation	2.19	(1.36–3.53)	0.001			
Chemotherapy	2.01	(1.17–3.44)	0.011	1.96	(1.14–3.38)	0.02
Any reconstruction	1.04	(0.63–1.71)	0.9			

ALND, axillary lymph node dissection.

### Adhesive Capsulitis

Adhesive capsulitis was associated with ipsilateral mastectomy, axillary lymph node dissection, and any reconstruction on univariate analysis; however, none of these variables were found to be significant in multivariate analysis, as shown in Table 5.

**Table 5.** Univariable and Multivariable Analysis of Adhesive Capsulitis following Mastectomy

Characteristic	Univariable			Multivariable		
	OR	OR 95% CI	*P*	OR	OR 95% CI	*P*
Any mastectomy	1.74	(0.68–4.44)	0.02			
Any node procedure	9.61	(9.60–9.63)	<0.001			
Any reconstruction	2.19	(1.02–4.74)	0.045			
Chemotherapy	0.2	(0.05–0.84)	0.03	0.16	(0.04– 0.63)	0.008

### Upper Extremity Musculoskeletal Dysfunction

Musculoskeletal dysfunction such as tendinopathy, arthritis, or rotator cuff injury were grouped together for analysis. Univariable analysis found musculoskeletal-based dysfunction was more likely in patients who did not have neoadjuvant chemotherapy or MRM as part of their treatment. Multivariable analysis identified no ipsilateral MRM (OR 0.17, CI 0.3–0.86, *P =* 0.033) to be associated with musculoskeletal dysfunction. Analysis results of variable association with upper extremity musculoskeletal dysfunction are shown in Table 6. Of the “other” types of dysfunction, there were no associated variables identified on further analysis.

**Table 6.** Univariable and Multivariable Analysis of Musculoskeletal Dysfunction following Mastectomy

Characteristic	Univariable			Multivariable		
	OR	OR 95% CI	*P*	OR	OR 95% CI	*P*
Ipsilateral cancer	0.69	(0.43–1.11)	0.13			
In situ cancer	1.27	(0.78–2.05)	0.3			
Invasive cancer	0.20	(0.05–0.74)	0.016			
MRM	0.15	(0.03–0.78)	0.024	0.17	(0.03–0.86)	0.033
Any reconstruction	0.64	(0.38–1.09)	0.10			
Chemotherapy	0.74	(0.42–1.30)	0.3			
Neoadjuvant chemotherapy	0.39	(0.18–0.83)	0.015			

MRM, modified radical mastectomy.

## DISCUSSION

Oncological extirpation remains the mainstay of breast cancer care. However, comprehensive treatment often includes chemotherapy and radiation therapy and, therefore, mastectomy does not usually occur in isolation. It is unclear, however, if these treatment modalities—each with different mechanisms of collateral trauma to the chest wall, shoulder girdle, and upper extremity—have a compounding effect. To this end, the authors conducted a retrospective chart review of 259 patients who underwent mastectomy and developed upper extremity dysfunction to determine if there are additional variables associated with pain, limited ROM, or other morbidity about the shoulder or arm. Specifically, we have demonstrated radiotherapy to be associated with ipsilateral upper extremity pain. Invasive cancer, any mastectomy, and radiotherapy are associated with ipsilateral limited ROM, and less-invasive surgery is associated with musculoskeletal dysfunction. We did not identify any variables associated with decreased strength, adhesive capsulitis, compressive neuropathy, or “other type” of upper extremity dysfunction on multivariable analysis. Any breast reconstruction was found to be associated with adhesive capsulitis, and implant reconstruction was found to be associated with limited ROM on univariable analysis, but neither were significant on multivariable analysis. Flap reconstruction was not found to be significantly associated with any type of dysfunction. There were no variables found to be significant for dysfunction of the contralateral extremity.

Radiation therapy was found to be associated with multiple cohorts of dysfunction. When the breast or axilla are irradiated, the chest wall, shoulder girdle, and upper extremity are often included within these fields. Shamley and colleagues have previously shown that radiotherapy is associated with lower activity in the pectoralis major, upper trapezius, and rhomboid muscles compared with mastectomy alone.^5^ Multiple studies have found radiotherapy to be associated with pain^22,23^ and limited ROM^22,24^ after surgery for breast cancer. Zarali et al^25^ found that, in patients treated with MRM or breast-conserving surgery, radiation is associated with arm stiffness but not reduction of strength, which is consistent with our findings. Interestingly, very few of the patients in their study had a diagnosis for upper extremity pathology outside their subjective complaints. Our results similarly show that radiotherapy was not associated with cohorts of dysfunction attributed to diagnoses such as adhesive capsulitis, nerve compression, and musculoskeletal pathologies, but rather impactful patient-reported symptoms.

Women who underwent MRM were less likely to develop musculoskeletal dysfunction on the side of surgery, regardless of reconstruction. An MRM is considered a more extensive surgery, involving removal of skin, breast tissue, a large number of axillary lymph nodes, and pectoralis muscle fascia. Most of the musculoskeletal pathologies found in our patient sample, such as tendonitis and rotator cuff injuries, may be associated with overuse. This finding may be a result of patients treated with MRM, limiting the use of their arm more than those following simple mastectomy, thus protecting themselves from an overuse injury.

Patients who did not receive neoadjuvant chemotherapy were more likely to have musculoskeletal dysfunction on univariable analysis. Although this finding may conflict with existing data on the effect of chemotherapy on the upper extremity, the majority of studies do not differentiate between neoadjuvant and adjuvant chemotherapy. Shamley et al^26^ found that any chemotherapy was associated with changes in scapulothoracic movement, whereas a recent meta-analysis showed any chemotherapy was associated with pain and decreased strength in patients after oncological breast surgery.^27^ Neoadjuvant chemotherapy is often associated with a reduction in tumor burden, and as such, perhaps patients who did not receive neoadjuvant chemotherapy and instead had upfront mastectomy underwent more aggressive operations such as MRM, which did prove to be significant on multivariable analysis.

Breast reconstruction including implant and flap subtypes were not found to be significantly associated with any cohort of upper extremity dysfunction on multivariable analysis. Of the analyzed variables, our findings surrounding the effect of reconstruction is contradictory to the current literature. Land et al^28^ showed patients receiving any type of reconstruction have increased subjective arm tightness compared with patients who underwent a mastectomy only. In contrast, Sun et al^29^ reported that patients receiving any type of breast reconstruction have reduced arm symptoms compared with patients who did not. Other studies evaluating dysfunction after autologous reconstruction report varying effects on the upper extremity as well. One study found that although arm pain is increased at 6-week and 6-month after latissimus dorsi flap reconstruction, at 1 year there is no difference in preoperative and postoperative pain.^30^ There are also data to support improved arm symptoms after other autologous reconstruction.^31^ In literature surrounding implant reconstruction, the data overwhelmingly show the positioning of the implant relative to the pectoralis major muscle to be linked to dysfunction, with subpectoral implants more associated with upper extremity dysfunction than prepectoral implants.^32–34^ Overall, the temporal relationship between breast reconstruction and upper extremity dysfunction is not well-defined; however, our data add to the evidence that it is not a major contributor to long-term dysfunction.

Although general evidence shows both sentinel and axillary dissection to be associated with upper extremity pain, mobility impairment, weakness, and sensory disturbance, nodal interventions were not found to be significant on multivariable analysis. However, any node procedure was significant on univariable analysis for multiple cohorts of dysfunction, and it may be that a larger sample size is needed for significance on multivariable analysis. Our findings that axillary node dissection was associated with dysfunction on univariable analysis while sentinel node biopsy was not significant at all is reflected in the literature, as multiple studies show axillary lymph node dissection to be more associated with upper extremity symptoms and functional loss than sentinel node biopsy.^35–38^

Although bilateral mastectomy was associated with limited ROM on univariable analysis, this was not true for multivariable analysis. Patients who underwent bilateral mastectomies in our study were on average 59 years old, whereas patients who underwent unilateral mastectomy were on average 66 years old. This younger age may contribute to improved mobility and recovery seen in our population. Prophylactic bilateral mastectomies have increased in popularity,^2^ and in our patient population 65 of 105 patients treated with bilateral mastectomies had cancer in only one breast, and nine of 105 patients treated with bilateral mastectomies were prophylactic bilaterally.

Limitations to the current study are largely based on the retrospective, single institution nature. Our sample size is limited by what is available in the patient charts and both common procedural technique and International Classification of Disease (ICD) codes to identify the original cohort. Our patient population may have experienced additional upper extremity symptoms that were not reported by the patient, documented, or diagnosed. ICD-10 codes were used to identify patients with upper extremity dysfunction, and there may have been patients who would otherwise meet inclusion criteria but whose dysfunction was not coded with the appropriate ICD-10 codes or those who may have sought care elsewhere, such as their primary physician. Some information of variables that may impact upper extremity function was not able to be collected given the retrospective nature, such as location, dosage, or boost of radiation therapy, or if the reconstruction was subpectoral or prepectoral. The authors purposefully excluded participants who developed lymphedema after mastectomy, as the weight of the arm from excess fluid and fibrosis has significant impact on the biomechanics of the upper extremity. We acknowledge that this is an imperfect review, but we feel it is valuable to inform future studies and protocols of early mobility or “prehabilitation.” Finally, this study did not include a cohort of patients treated with a mastectomy who did not develop any upper extremity dysfunction. A follow-up study comparing a case-matched control cohort with the current affected population of this study will provide additional insight to those at risk for upper extremity dysfunction.

## CONCLUSIONS

As mastectomy with or without reconstruction continues to be a mainstay of breast cancer treatment, surgical technique and enhanced recovery pathways have been designed to improve outcomes and minimize complications. It is necessary to assess the impact of these treatments on long-term quality of life, including pain, mobility, and function of the upper extremity. Our study identifies statistically significant risk factors of same-sidedness and radiation therapy predicting upper extremity dysfunction. Breast reconstruction did not have an effect on long-term dysfunction. Although neoadjuvant chemotherapy seemed to be protective, further studies will need to be conducted to fully understand this observation. The current data support physical rehabilitation for patients with the identified characteristics and inform future prospective studies investigating the role of early mobility or prehabilitation in comprehensive breast cancer care.

## DISCLOSURES

The authors have no financial interest to declare in relation to the content of this article. This study was funded in part by NIH/NCI (grant no.: R25CA240134).
